# Peripheral Neuropathy in Patients with Hepatitis C Infection—Reversibility after HCV Eradication: A Single Center Study

**DOI:** 10.3390/v16040522

**Published:** 2024-03-28

**Authors:** Theodoros Androutsakos, Ioanna Tsantzali, Dimitrios S. Karagiannakis, Pagona Flevari, Despoina Iakovou, Abraham Pouliakis, Stylianos Kykalos, Stylianos Doris, Vasileia Xyla

**Affiliations:** 1 Department of Pathophysiology, National and Kapodistrian University of Athens, 115 27 Athens, Greece; siliaxyla@yahoo.gr; 2 Second Department of Neurology, School of Medicine, National and Kapodistrian University of Athens, “Attikon” General University Hospital, 124 62 Athens, Greece; docjo@gmail.com; 3 Academic Department of Gastroenterology, Laiko General Hospital, National and Kapodistrian University of Athens, 115 27 Athens, Greece; dkarag@med.uoa.gr; 4 Centre of Excellence in Rare Haematological (Haemoglobinopathies) & Rare Metabolic (Gaucher Disease) Diseases, Laiko General Hospital, 115 27 Athens, Greece; fpagona@yahoo.gr; 5 West Suffolk Hospital NHS Foundation Trust, Bury St Edmunds IP33 2QZ, UK; iakovoudespina@gmail.com; 6 Second Department of Pathology, National and Kapodistrian University of Athens, 124 62 Athens, Greece; apou1967@gmail.com; 7 Second Department of Propaedeutic Surgery, National and Kapodistrian University of Athens, 115 27 Athens, Greece; kykalos@gmail.com; 8 Neurology Department, Metropolitan General Hospital, 155 62 Athens, Greece; steliosdoris@gmail.com

**Keywords:** HCV, DAA, peripheral neuropathy, nerve biopsy, electroneurography

## Abstract

Chronic hepatitis C virus (HCV) infection is characterized by a variety of extra-hepatic manifestations; peripheral neuropathy (PN) is one of the most common, especially when mixed cryoglobulinemia (MCG) is present. The prevalence and risk factors of HCV-related PN in the absence of MCG are largely unknown. We conducted a prospective, single-center study, examining the prevalence and reversibility of HCV-associated neuropathy in the absence of MCG. Nerve fiber density in the epidermis was evaluated through skin biopsy and electroneurography (ENG) before HCV-treatment initiation and 1 year post sustained virological remission (SVR). Forty HCV-infected individuals (nine HIV co-infected) with no other neuron-harming factors were included; four other HCV mono- and three HIV co-infected individuals were excluded due to presence of diabetes, B12 insufficiency, or neurotoxic drugs. Twelve consecutive controls with no neuron-harming conditions were also recruited; eight more were excluded due to meeting exclusion criteria. Four patients had ENG signs of polyneuropathy (two with HCV mono- and two with HIV co-infection), while seven more (five with HCV mono- and two with HIV co-infection) had signs of mono-neuropathy, leading to PN prevalences of 22.5% and 44% for mono- and co-infection, respectively (*p* value 0.179). The two patients with HCV mono-infection and polyneuropathy and the one with ulnar nerve damage showed ENG improvement 1 year post SVR. Regarding intraepidermal nerve density, HCV infection, irrespective of HIV co-infection, was correlated with a lower intraepidermal neuron density that improved 1 year post SVR (*p* value 0.0002 for HCV and 0.0326 for HCV/HIV co-infected patients). PN is common in HCV infection; successful eradication of HCV leads to PN improvement.

## 1. Introduction

Hepatitis C virus (HCV) is a single-stranded positive-sense RNA virus that belongs to the family of Flaviviridae and is the sole member of the genus Hepacivirus [1]. Chronic HCV infection constitutes a major global health issue, with more than 70 million patients being infected worldwide and at least 400,000 deaths due to HCV occurring each year [2,3]. However, the landscape of chronic HCV infection treatment has changed substantially in the last decade due to the emergence of direct-acting antiviral drugs (DAAs), which are associated with over 95% of sustained virological responses (SVRs) in all patients, irrespective of the presence of cirrhosis, comorbidities, or genotype [4,5,6].

Chronic HCV infection is characterized by a variety of extrahepatic manifestations (EHMs). These EHMs can be further classified as organ specific (including thyroid, pulmonary, renal, dermatological, and ocular), general (mainly chronic fatigue), cardiovascular, metabolic, neuropsychiatric, lymphoproliferative, and autoimmune [7,8,9]. Peripheral neuropathy (PN) is a common EHM in HCV-infected individuals, varying in prevalence from 26 to 86% when mixed cryoglobulinemia (MCG) is present [10,11,12]. The exact pathogenesis of nerve injury in HCV infection is largely unknown; however, it is believed to be secondary to two main pathogenetic mechanisms: on one hand, the alteration of microcirculation of the vasa nervorum due to the intravascular deposit of cryoglobulins and consequent vessel obstruction and ischemia, and on the other hand, necrotizing vasculitis induced by longstanding precipitation of immune complexes composed of substances like cryoglobulins, activated complement, rheumatoid factor, and viral proteins [13,14,15,16,17]. PN in the clinical course of HCV has been mainly examined in conjunction with cryoglobulinemia; on the contrary, only a handful of studies have examined it when cryoglobulins are absent [18,19,20,21,22,23,24]. The aim of this study is to examine the prevalence and reversibility of PN in HCV-infected individuals before and after successful viral eradication with DAAs.

## 2. Materials and Methods

### 2.1. Inclusion, Exclusion Criteria, Testing, and Follow Up

Consecutive HCV-infected patients (defined as patients with detectable HCV RNA in their peripheral blood) referred for treatment to the outpatient hepatology clinic of “Laiko” General Hospital of Athens, Greece, were eligible for recruitment. Inclusion criteria were age >18 years and signed informed consent, while exclusion criteria were the presence of diabetes mellitus, low serum B12, cryoglobulinemia, liver disease other than HCV, hepatic or extrahepatic cancer, as well as patients’ inability to receive treatment (due to poor compliance, concurrent alcoholism, or active intravenous drug use). Healthy individuals referred to the hepatology department for evaluation of various diseases, like liver hemangiomas or cysts, with no exclusion criteria met, according to the study protocol, were used as control group.

Written consent was acquired from all study participants; this study complied with the principles of the Declaration of Helsinki and the general data protection rules (GDPRs) of the European Union, and it was approved by the Ethics Committee of “Laikon” General Hospital (protocol number 14492/15-10-2018). 

Medical history was obtained through patient interviews and their medical charts. The duration of HCV infection was defined as the duration between patients’ HCV diagnosis (via PCR assessment of HCV viral load) and their enrollment day. All patients underwent transient elastography (TE) before DAA initiation and were deemed to be at high or low risk for advanced chronic liver disease (ACLD) using a TE cut-off of 10kPa, according to the latest EASL guidelines [25]. Thorough laboratory testing (including, among others, serum transaminases, serum total, high and low-density cholesterol and serum triglycerides levels, glycosylated hemoglobin, and glucose levels), thorough neurological examination, and skin biopsy were performed upon study inclusion, while an electroneurography (ENG) was performed within 1 week of inclusion. Type 2 diabetes mellitus (T2DM) was defined as concurrent antidiabetic treatment or levels of blood sugar ≥ 126 mg/dL or ≥200 mg/dL for fasting and non-fasting patients, respectively, or glycosylated hemoglobin of (HbA1c) ≥ 6.5% [26]. Fibrosis-4 (FIB-4) scores and aspartate aminotransferase (AST) to platelet ratio (APRI) scores were calculated from patients’ laboratory results, as explained in the relevant references [27,28]. A cut-off of 1.3 and 1.5 were used to identify patients at high risk of ACLD, according to the EASL guidelines [25]. 

All the HCV-infected patients were treated with DAAs according to national and international guidelines [29] and were followed by the outpatient hepatology clinic as per usual practice. Sustained virological response (SVR) was defined as the absence of HCV RNA in a patient’s blood 3 months after DAA treatment, according to guidelines [29]. One year post SVR, a new neurological examination, skin biopsy, laboratory testing, and ENG were performed. Patients with no HCV infection that did not meet the exclusion criteria were included as healthy controls, while patients with human immunodeficiency virus (HIV) and HCV co-infection also treated with DAAs were defined as the disease control group. All skin biopsies were evaluated by the same experienced personnel, while all EMGs were performed by the same expert neurologist.

### 2.2. Skin Biopsy Staining and Evaluation

For the evaluation of PN, the quantification of nerve fiber density in the epidermis with the use of the protein gene product (PGP 9.5) stain [30] was conducted. PGP 9.5 is a pan-axonal marker that visualizes unmyelinated C-fibers and small myelinated Ad fibers [31,32] at the epidermis, papillary, and reticular dermis; blood vessels; exocrine sweat glands; and hair follicles [33].

A skin biopsy from each patient was obtained from the lower calf, 10–20 cm above the lateral malleolus, with a 4 mm punch device, and then formalin-fixed and paraffin-embedded. This site was used as a typical site of skin biopsy for the evaluation of PN due to its proximity to the sural nerve, its high repeatability, and the lack of adverse events [34,35]. Following de-paraffination, each biopsy specimen was cut, and 8 sections of 15 μm thickness were obtained. Immunohistochemical staining was performed manually using a PGP 9.5 polyclonal antibody marker (Ultraclone, Histon, Cambridgeshire, UK) with respect to the manufacturer’s recommendations for antigen retrieval [36]. All 8 sections from each specimen were examined for intraepidermal nerve fiber density by two independent pathologists who used a simple counting and calculating method under light microscopy at high magnification. For calculation of the number of intraepidermal nerve fibers, we followed the method described in previous works from our department and others [37,38,39]. For each patient, the final value entered in the analysis was the median value of the 8 measurements. Nerve fiber density was recorded as the number of nerve fibers/mm. The length and the surface area of the epidermis of each specimen were measured by using image analysis software (SigmaScan v. 2.0, Jandel Scientific, Erkrath, Germany) on a Unisys CWP 5753 PC (Unisys Corp., San Jose, CA, USA).

### 2.3. Electroneurography

For the neurophysiological evaluation of peripheral neuropathy, all patients underwent ENG within 1 week from the study inclusion date after the diagnosis. The test was repeated twelve months post SVR to assess any affection of the peripheral nerves. The ENG included a motor conduction study and F-wave of the median, ulnar, peroneal, and tibial nerves bilaterally and a bilateral sensory conduction study of the median, ulnar, and sural nerves. Stimulation and detection were performed by using superficial electrodes. The sensory conduction study was performed by using antidromic stimulation.

### 2.4. Statistical Analysis

Statistical analysis was performed via the SAS for Windows 9.4 software platform (SAS Institute Inc., NC, USA). Descriptive values for the arithmetic data were expressed as medians and ranges (i.e., minimum and maximum values), and for the categorical data were presented as frequencies and relevant percentages. Comparisons between the groups for the qualitative parameters were performed via the chi-square test and, when required, via Fisher’s exact test. For the arithmetic data (such as age or laboratory test results), since it was not possible to ensure normality using the Shapiro–Wilk test, non-parametric tests were applied; specifically, for comparisons between two categories, the Mann–Whitney U test was applied, and for more than two categories, the Kruskal–Wallis test was used; for paired tests, the Wilcoxon signed rank test or the sign test (when data were limited) were applied. Furthermore, to evaluate for possible factors that may be related to an abnormal ENG, we applied univariable analysis, and to control for possible confounders, we applied logistic regression using selected variables such as age, gender, and number of intraepidermal fibers as input variables. Finally, we evaluated correlations in the number of intraepidermal fibers at baseline with all the laboratory results using Spearman correlation coefficients, and we also applied a multiple linear regression analysis. The significance level (*p*-value) for all study tests was set to <0.05, and all tests were two-sided.

## 3. Results

### 3.1. Demographic and Laboratory Values

During the study period, a total of 35 HCV-infected and 12 HCV/HIV-co-infected patients were screened for inclusion. Of them, three HCV and two HCV/HIV patients suffered from diabetes mellitus, while one HCV/HIV individual suffered from lymphoma and one from multiple myeloma; they were thus excluded. Regarding the healthy individual group, 8 out of the 20 recruited patients were excluded due to the presence of diabetes mellitus. As a result, 31 HCV-infected (86.1% of all HCV patients), 9 HCV/HIV-co-infected (81.8% of all HCV/HIV patients), and 12 healthy individuals fulfilled the inclusion criteria and were enrolled in this study. The demographic and laboratory examination values of these patients are presented in Table 1. Overall, the patients with HCV/HIV co-infection had a shorter duration of HCV infection and higher serum alanine aminotransferase (ALT) and aspartate aminotransferase (AST) levels, while the controls were older than the patients with HCV mono- or co-infection, also including a higher percentage of females (Table 1). As expected, the HCV-infected individuals exhibited higher transient elastography results; four individuals in the mono- and three in the co-infected groups had TE values of more than 10 kPa and were deemed to be at high risk of ACLD, according to their TE results, while according to the FIB-4 and APRI scores, the number of patients at risk of ACLD was 11 out of 31 (35.5%) and 8 out of 31 (25.8%) for the HCV mono-infected patients, respectively, and 5 out of 9 (55.5%) and 1 out of 9 (11.1%) for the HCV/HIV co-infected patients, respectively (Table 1). Genotypes 1 and 3 were most commonly found in the mono- and co-infected individuals, respectively.

### 3.2. Neural Biopsies

**Figure 1:**
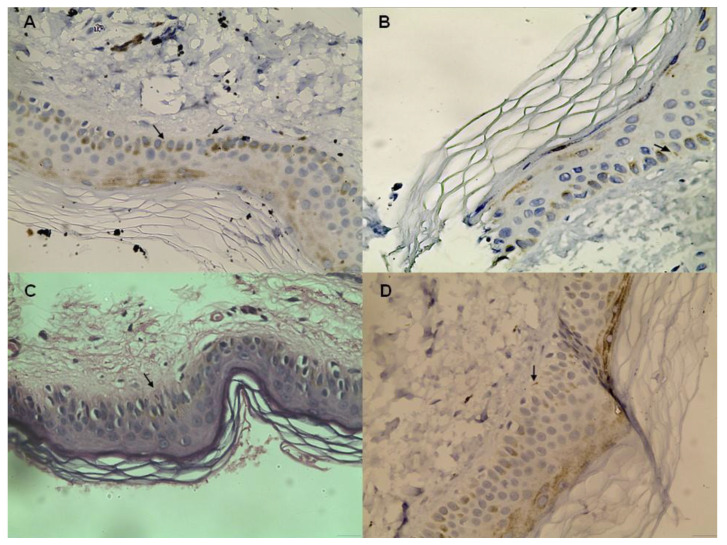
Immunostaining for PGP 9.5 in the epidermis from a control subject (**A**), a patient with HCV/HIV coinfection (**B**), and a HCV patient before (**C**) and after (**D**) treatment. Arrows showing epidermal nerve fibers.

**Figure 2:**
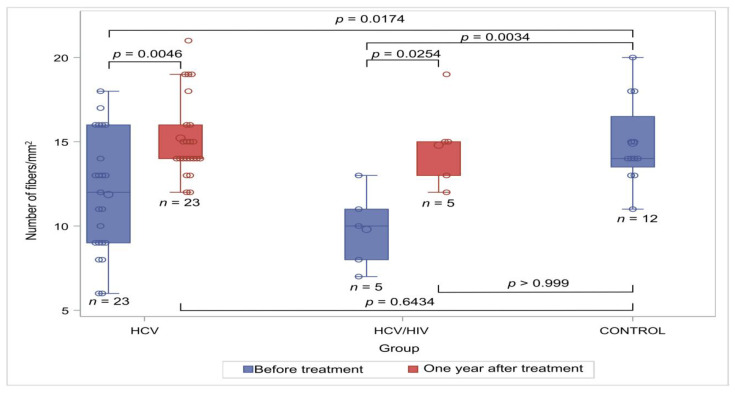
Number of intraepidermal fibers/mm^2^ in the study groups. Small circles correspond to data points and larger circles correspond to the median value. Abbreviations: HCV: hepatitis C virus; HIV: human immunodeficiency virus.

A total of 40 HCV-infected individuals, including 9 with HIV co-infection, underwent a skin biopsy at baseline, and 33, including 8 with co-infection, underwent post-DAAs treatment. Typical immunostaining figures from the four groups can be found in Figure 1. The median time between the first and second biopsy among patients with both HCV mono- and co-infection was 17 months (range 12–22). The median number of neuronal fibers/mm^2^ in each one of these groups as well as that of controls is presented in Table 2 and Figure 2. All the HCV-infected patients showed a lower density of intraepidermal nerves when compared to the controls; this difference reached statistical significance in all three groups before treatment (*p* = 0.0067 when excluding patients lost to follow up or with missing data from the analysis). Neuron density after treatment was similar to that of the control group in both the HCV mono- and HCV/HIV-co-infected individuals (*p* = 0.6434 and *p* > 0.999, respectively).

### 3.3. Electroneurography Results

A total of seven patients with HCV mono-infection exhibited an abnormal ENG; two showed a moto-sensory polyneuropathy, while three had an abnormal peroneal, one tibial and one ulnar nerve wave. Interestingly enough, the two patients with moto-sensory polyneuropathy showed an improvement in ENG 1 year post SVR; likewise, the patient with the tibial nerve neuropathy presented with a normal ENG upon his follow up; for the other patients, no improvement was noted. 

Regarding the HCV/HIV-co-infected patients, moto-sensory polyneuropathy was noted in two patients, while in two more, ulnar neuropathy was discovered; no improvement after HCV eradication was noted in any of them.

### 3.4. Characteristics of Patients with Peripheral Neuropathy

**Figure 3:**
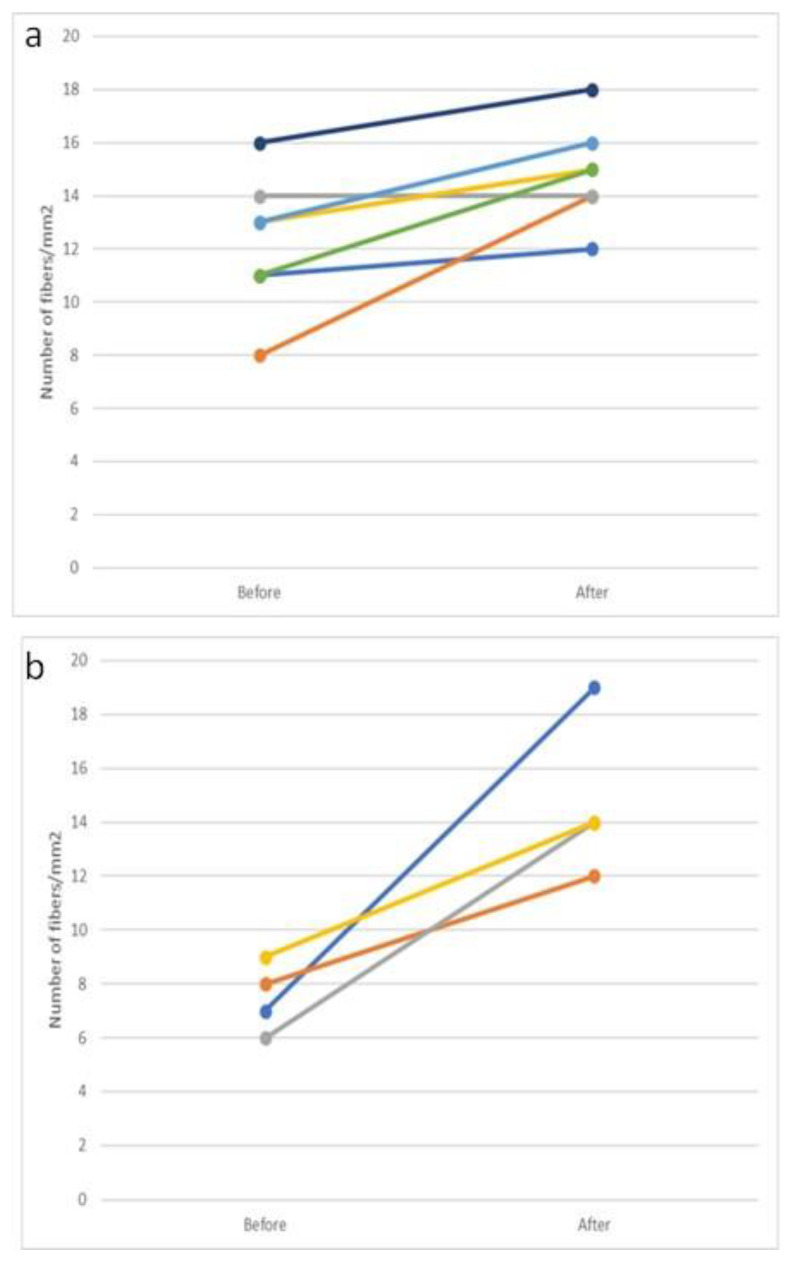
Number of intraepidermal fibers/mm^2^ (**a**) for the seven patients with peripheral mono-neuropathy before and after treatment and (**b**) for the four patients with moto-sensory polyneuropathy before and after treatment. Each color presents different patient.

The four patients with moto-sensory polyneuropathy were all males, with a median age of 49 years (range 32–61). The median levels of serum HCV RNA were 2,695,000 cop/mL and the median duration of HCV infection was 12.5 years (range 7–28); HCV genotype 3 was found in three of the patients and genotype 4 was found in the other one. All four patients showed an improvement in intraepidermal nerve density; the median intraepidermal nerve density before treatment was 6.5 (range 5–9) and 1 year post SVR was 14 fibers/mm^2^ (range 12–19) (*p* = 0.0273) (Figure 3a). 

Regarding the patients with peripheral mono-neuropathy, five out of seven patients were male; two of them (one male and one female) were HIV co-infected. The median age of these patients was 41 years (range 32–44), the median serum HCV RNA level 1,559,000 cop/mL, and the median duration of HCV infection was 3 years (range 1–15); HCV genotype 1 was found in three, genotype 3 in two, and genotype 4 in one of the patients. Five patients showed an improvement in nerve density measurement after DAA treatment; however, only in one of them was that improvement significant (Figure 3b).

### 3.5. Risk Factors for Abnormal ENG

The patients with abnormal ENG presented with higher values of serum direct bilirubin and gamma glutamyl-transferase, while no differences were noted regarding gender, age, HCV genotype, HCV duration, and fibrosis staging (Table 3). As expected, a higher density of intraepidermal fibers was related to a higher probability of normal ENG, even though this finding did not reach statistical significance (Supplementary Figure S1). 

### 3.6. Factors Affecting Intraepidermal Nerve Density

In the univariable analysis, female gender (*p* = 0.0233) was correlated with a higher presence of HCV infection (*p* = 0.0175) and with a lower intraepidermal nerve density (Table 4A). 

**Figure 4:**
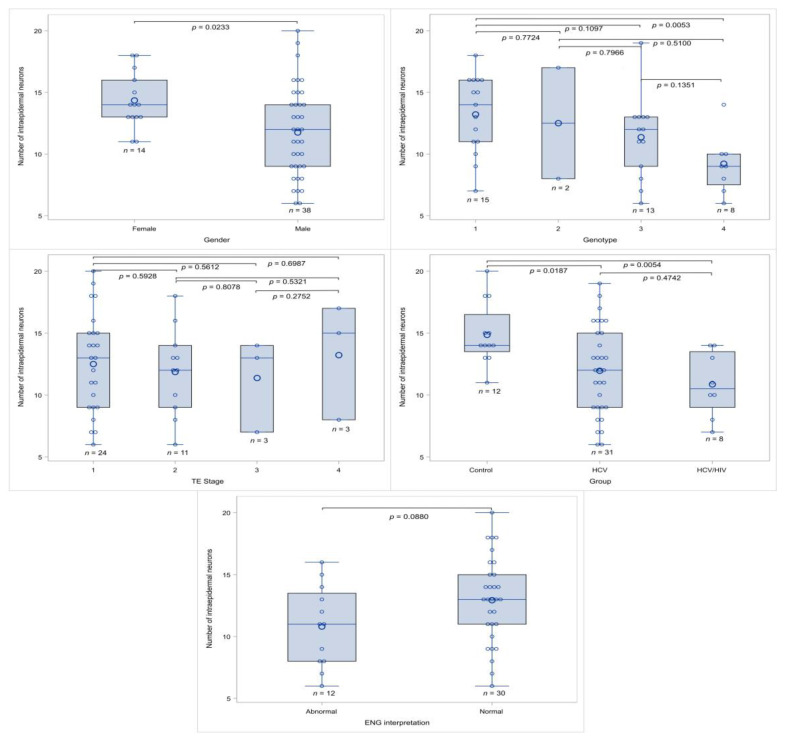
Box-and-whisker plots for intraepidermal nerve density with the study characteristics. Small circles correspond to data points and larger circles correspond to the mean value.

Patients with abnormal ENG had lower neural density; however, in our study cohort, this was marginally significant (*p* = 0.0880). Characteristics such as patients’ age, HCV RNA and duration, laboratory blood results, and outcomes of scoring systems were evaluated via the Pearson correlation coefficient, and in no case was a significant correlation found (Table 4A). Characteristic box-and-whisker plots for the number of intraepidermal nerves in relation to the study characteristics are shown in Figure 4.

In a multivariable approach, we exercised several scenarios (a) for the role of gender, age, and HCV vs. non HCV at baseline; (b) for the gender, age, and HIV vs. non HIV at baseline; and (c) for the role of gender, genotype (examined as genotype 1 vs. genotype 2–4 combined), staging (examined as F0–3 vs. F4), and timing (i.e., before and after treatment) to examine their role in intraepidermal nerve density measurements.

Patients with HCV at baseline had a lower nerve density (*p* = 0.0354), while female gender was correlated with a higher intraepidermal nerve density measurement. The presence of genotype 1 was marginally correlated with a higher nerve density when compared with genotypes 2–4 (*p* = 0.0417). Moreover, successful treatment was also positively correlated with a higher neural fiber count; however, this finding did not reach statistical significance (*p* = 0.1321) (see Table 4B). Fibrosis staging or HIV co-infection were not correlated with epidermal nerve density.

## 4. Discussion

PN is a relatively common EHM of chronic HCV infection; however, its exact prevalence and pathogenesis in the absence of MCG are largely unknown. HCV-related neuropathies include symmetrical axonal sensorimotor neuropathy, accounting for over 50% of cases; distal symmetric painful small-fiber neuropathy, with predominantly sensory features; mononeuritis multiplex; pure motor polyneuropathy; or, rarely, demyelinating and autonomic neuropathies, leading to a reduced quality of life [10,40,41]. The introduction of an all-oral DAA treatment for HCV has led to impressive results regarding viral eradication; however, the impact on HCV-related EHM is still in doubt, even though most studies have revealed an ameliorating effect [42,43,44,45]. We conducted a prospective, single-center study to estimate the prevalence and impact of HCV eradication in HCV-associated neuropathy in the absence of MCG. 

Our cohort consisted of 40 HCV-infected individuals (nine of them with an HIV co-infection) with no history of diabetes mellitus, B12 deficiency, alcohol overuse, or other known neuron-harming factors. A total of 4 out of 40 patients in our cohort exhibited signs of polyneuropathy (2 with mono- and 2 with co-infection), while 7 more patients (5 with mono- and 2 with co-infection) showed ENG signs of mono-neuropathy, leading to a PN prevalence of 22.5% and 44% for mono- and co-infection, respectively. In a large study from Cacoub P et al., comprising 86 patients with HCV infection and no cryoglobulinemia, PN was reported by 8%; however, PN was only assessed via questionnaire [11]. In another large study by Zanone M et al., PN was noted in a significantly higher percentage (22%) that is close to ours; this time, PN was assessed by ENG [46]. In our study, post treatment was normal in three out of the seven patients with abnormal ENG and HCV mono-infection ENG; on the contrary, no patients with HIV co-infection showed improved ENG findings. Likewise, Zanone M et al. have shown an improvement in ENG findings after DAA treatment, even though most of their measurements did not reach statistical significance [46]. A possible explanation could be the relatively small number of patients with PN in both cohorts.

In our cohort, ENG findings were unrelated to the duration of HCV infection or fibrosis staging. Regarding HCV duration, current data are largely conflicting. In a study by Biassiotta A et al., HCV duration and age, in contrast to the presence of cryoglobulinemia, were predisposing factors for PN; on the contrary, in a study by Niemni R et al., the presence of cryoglobulinemia was a key factor for the presence of PN [18,21]. A possible explanation for our findings could be the younger age of our patients and the smaller duration of HCV infection when compared with those in the study by Biassiotta et al. (42 vs. 69 and 7.5 vs. 16 years, respectively). 

As far as intraepidermal nerve density is regarded, HCV infection, irrespective of the presence of HIV co-infection, was correlated with a lower intraepidermal neuron density in our cohort, highlighting the harmful effect of HCV in nervous tissue. To further highlight this role, intraepidermal neuron density rose 1 year after DAA treatment. Since no studies have shown neuronal activity amelioration with the use of DAAs, this result should be attributed to HCV eradication. To our knowledge, no other studies have examined the effect of DAA treatment on intraepidermal neuron density after HCV eradication. Interestingly enough, higher intraepidermal nerve density was correlated with female gender and HCV genotype 1. The positive effect of female gender in epidermal nerve density has already been reported in previous studies, although this finding is not unequivocally accepted [47,48,49]. Regarding the effect of HCV genotype 1 on peripheral nerve damage, our findings come to disagreement with those of Zanone M et al. [49], and they must surely be interpreted with caution as the number of patients in each subgroup is low.

Our study has certain strengths and limitations. The long duration of the study and the evaluation of PN with both skin biopsy and ENG in the same patients before and after DAA treatment, as well as the exclusion of patients with MCG, are the main strengths of this study; on the other hand, the small cohort, especially in regard to patients with HIV co-infection and those with cirrhosis, and the fact that our study does not add any knowledge to the mechanisms underlying neurological improvement, are the main limitations.

## 5. Conclusions

Overall, we show that the successful eradication of HCV with DAAs leads to an improvement in PN, both histologically and through nerve conduction studies. Extended multicenter studies, possibly with a longer follow up duration, are needed to further confirm our findings. 

## Figures and Tables

**Table 1:** Baseline demographic and laboratory values among 3 groups.

	HCV Group (*n* = 31) Median (Range)	HCV/HIV Group (*n* = 9) Median (Range)	Healthy Controls (*n* = 12) Median (Range)	*p* Value
Age	43 (29–64)	41.5 (32–52)	57.5 (31–71)	**0.0356**
Male *n*, %	25 (80.7%)	8 (88.9%)	5 (41.7%)	**0.0281**
Duration of HCV infection in years	9 (1–28)	7 (1–11)	n/a	0.3823
HCV genotype 1/2/3/4	13/2/10/6	3/0/3/2	n/a	0.0729
HCV RNA in IU/mL	1,500,000 (110,000–37,000,000)	3,400,000 (120,000–6,456,000)	n/a	0.5819
Serum AST value	35 (15–164)	54 (21–161)	25 (10–42)	**0.0023**
Serum ALT value	52 (17–233)	74 (21–301)	31.5 (12–41)	**0.0006**
Serum ggt value	35 (9–198)	61 (30–160)	28.5 (9–60)	**0.02041**
Serum ALP value	67 (28–272)	80 (66–147)	67.5 (34–261)	0.0955
PLTs value	210,000 (118,000–452,000)	169,000 (156,000–329,000)	275,000 (120,000–401,000)	0.1454
TE value	7.7 (3.5–35.5)	9.3 (4–12)	5.6 (3.2–8.3)	0.1889
TE value (≥10/<10)	4/23	3/3	0/9	**0.0426**
Fib-4 score	1.1 (0.2–4.5)	1.5 (0.5–2.3)	0.9 (0.3–3)	0.4397
Fib-4 score (≥1.3/<1.3)	11/20	5/4	3/9	0.4021
APRI score	0.6 (0.1–8.7)	1 (0.3–2.3)	0.3 (0.1–0.9)	**0.0023**
APRI score (≥1.5/<1.5)	8/23	1/8	0/12	0.1066

Results in bold show statistical significance. Abbreviations: HCV: hepatitis C virus; n/a: not applicable; HIV: human immunodeficiency virus; AST: aspartate aminotransferase; alanine aminotransferase; ggt: gamma glutamyl-transferase; ALP: alkaline phosphatase; TE: transient elastography; PLTs: platelets; Fib-4: fibrosis-4; APRI: AST to platelet ratio.

**Table 2:** Values of intraepidermal nerve density in the study groups after the exclusion of patients lost to follow up or with missing data.

	HCV Group before Treatment (*n* = 31)	HCV Group after Treatment (*n* = 25)	HCV/HIV Group before Treatment (*n* = 9)	HCV/HIV Group after Treatment (*n* = 8)	Healthy Controls (*n* = 12)	*p* Value
Number of neuronal fibers/mm^2^ in upper epidermal layer, median (range) after exclusion of patients lost to follow up	12 (6–18)	14 (12–21)	10 (7–13)	15 (12–19)	14 (11–20)	0.0067 *

Abbreviations: HCV: hepatitis C virus; HIV: human immunodeficiency virus. *: before treatment and among the three groups.

**Table 3:** Univariable analysis for ENG.

	ENG Outcome		
	Abnormal	Normal	
Characteristic	Median (Range) or Number of Cases	Median (Range) or Number of Cases	*p* Value
Age	42 (35.5–52)	39.5 (34–53.5)	0.8298
Gender (male)	11/91.67%	16/76.19%	0.3792
Genotype (1/2/3/4)	4/0/6/2	10/2/6/4	0.5997
HCV duration (years)	7.5 (2–13.5)	8 (1–18)	0.8031
Number of intraepidermal neurons/mm^2^	11 (8–13.5)	12 (9–13.5)	0.4113
HCV RNA (IU/mL)	1,953,585 (612,000–4,829,705)	1,860,000 (580,000–4,530,000)	0.7933
Infection (HCV/HCV and HIV)	8/4	18/3	0.3774
AFP (ng/mL)	4 (2.8–5.5)	3 (2–4)	0.0803
ALP (IU/L)	67.5 (65–88)	67 (53–79)	0.5872
ALT(IU/L)	56 (37.5–161.5)	63 (36–101)	0.6532
AST(IU/L)	43.5 (24–112.5)	35 (27–64)	0.7361
Direct bilirubin (mg/dL)	0.5 (0.3–0.7)	0.3 (0.2–0.5)	**0.0430**
Total bilirubin (mg/dL)	0.87 (0.7–1.2)	0.8 (0.5–1)	0.1237
ggt (IU/L)	69.5 (29.5–114)	33 (21–49)	**0.0454**
HbA1C (%)	5.25 (5–6)	5.35 (5.1–5.7)	0.9151
INR	1 (1–1.1)	1 (1–1)	0.2140
PLTs (k/mL)	176,000 (160,500–260,500)	231,000 (177,000–246,000)	0.5493
APRI	0.84 (0.3455–1.62)	0.606 (0.424–1.124)	0.9106
APRI score (≥1.5/<1.5)	3/9	4/17	0.6856
FIB-4	1.27 (0.69–2.085)	0.99 (0.61–1.29)	0.3691
Fib-4 score (≥1.3/<1.3)	6/6	5/16	0.1490
TE score	6.6 (4.3–9.2)	7.7 (4.35–8.35)	0.8951
TE value (≥10/<10)	2/8	2/14	0.6254

Results in bold show statistical significance. Abbreviations: ENG: electroneurography; HCV: hepatitis C virus; AFP: alpha fetoprotein; ALP: alkaline phosphatase; ALT: alanine aminotransferase; AST: aspartate aminotransferase; ggt: gamma glutamyltransferase; HbA1C: hemoglobin A1C; INR: international normalized ratio; PLTs: platelets; APRI: AST to platelet ratio index; FIB-4: fibrosis-4 index; TE: transient elastography.

**Table 4:** Univariable and multivariable analysis results for the intraepidermal nerve density levels *: Wilcoxon signed-rank test for the patients before and one year after treatment was applied. (**A**) Univariable analysis. (**B**) Multivariable analysis models.

**(A)**				
**Variable**	**Values**	**Fiber Count (Median (Range))**		***p* Value**
Gender	F	14 (11–18)		**0.0233**
	M	12 (6–20)		
Group	Control	14 (11–20)		**0.0175**
	HCV	12 (6–19)		
	HCV/HIV	10.5 (7–14)		
Genotype	1	14 (7–18)		**0.0476**
	2	12.5 (8–17)		
	3	12 (6–19)		
	4	9 (6–14)		
Staging	F0–1	13 (6–20)		0.8343
	F2	12 (6–18)		
	F3	13 (7–14)		
	F4	15 (8–17)		
ENG	Abnormal	11 (6–16)		0.0880
	Normal	13 (6–20)		
TE value	≥10	13.5 (7–17)		0.9557
	<10	13 (6–20)		
Treatment *	Before treatment	13 (10–15)		**0.0012**
	One year after SVR	14.5 (14–16)		
**(B)**				
	**Parameter**	**Beta**	**Standard Error**	***p* Value**
Model a	Gender (female)	1.57	1.11	0.1652
	Age	-0.01	0.04	0.7586
	Group HCV (ref non-HCV)	-2.67	1.23	**0.0354**
Model b	Gender female	2.26	1.09	**0.0439**
	Age	0.01	0.04	0.8601
	Group HIV (ref non-HIV)	-1.46	1.34	0.2804
Model c	Gender female	3.81	1.4	**0.0110**
	Genotype 1 (reference: genotype 2–4)	2.51	1.18	**0.0417**
	Staging F4 (reference: stage not 4)	1.3	1.69	0.4500
	Treatment (reference: before treatment)	3.63	2.35	0.1321

Abbreviations: ENG: electroneurography; TE: transient elastography; F: female; HCV: hepatitis C virus; HIV: human immunodeficiency virus; M: male; SVR: sustained virological response.

## Data Availability

All data regarding this article are available upon reasonable request.

## Supplementary Materials

The following supporting information can be downloaded at: https://www.mdpi.com/article/10.3390/v16040522/s1, Figure S1: Analysis results of analysis for the probability of abnormal ENG.
