# Supplementary material for: Peripheral Neuropathy in Patients with Hepatitis C Infection—Reversibility after HCV Eradication: A Single Center Study
Source: Viruses. 2024 Mar 28;16(4):522. doi: 10.3390/v16040522 (PMC11054011; doi:10.3390/v16040522)
Supplement: Supplementary file 1 [file viruses-16-00522-s001.zip › viruses-2874577-supplementary.pdf]

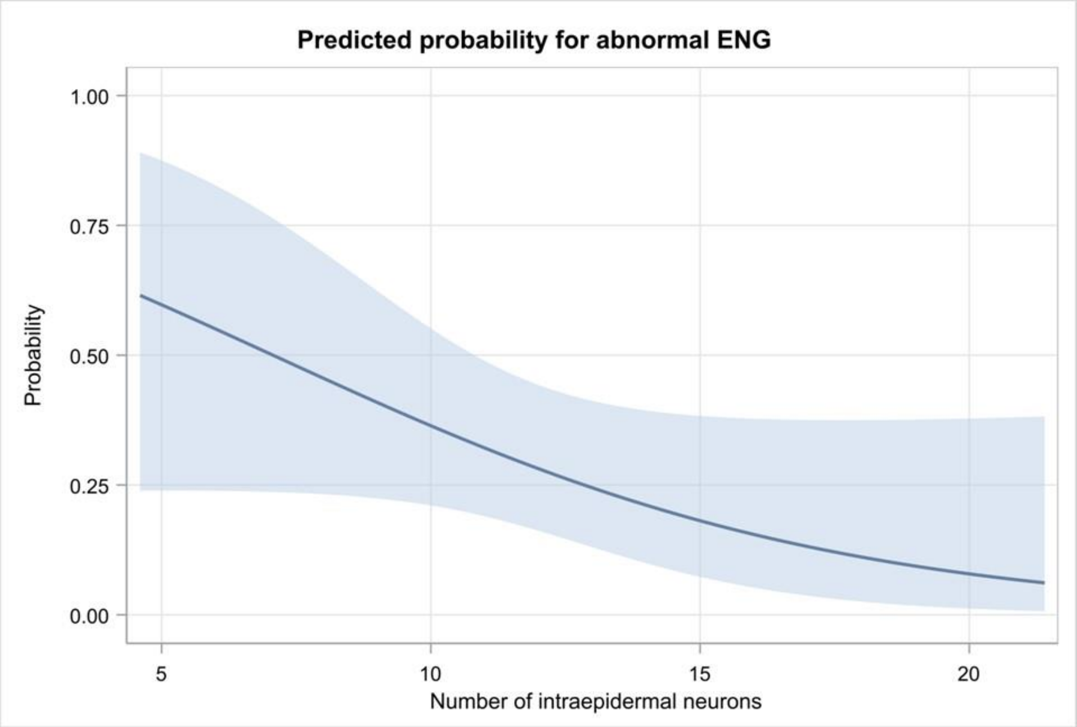

Figure S1. Analysis results of analysis for the probability of abnormal ENG. The shaded area indicates the 95% confidence interval for the probability for abnormal ENG prediction.
